# Single-Night Sleep Extension Enhances Morning Physical and Cognitive Performance Across Time of Day in Physically Active University Students: A Randomized Crossover Study

**DOI:** 10.3390/life15081178

**Published:** 2025-07-24

**Authors:** Eya Bouzouraa, Wissem Dhahbi, Aymen Ferchichi, Vlad Adrian Geantă, Mihai Ioan Kunszabo, Hamdi Chtourou, Nizar Souissi

**Affiliations:** 1 Research Unit “Sport Sciences, Health and Movement”, High Institute of Sports and Physical Education of Kef, University of Jendouba, Kef 8100, Tunisia; eya.bouzoraa@gmail.com (E.B.); wissem.dhahbi@gmail.com (W.D.); aymenferchichi2020@gmail.com (A.F.); 2 Training Department, Police College, Qatar Police Academy, Doha 59J5+C7R, Qatar; 3 Faculty of Physical Education and Sport, Aurel Vlaicu University of Arad, 310330 Arad, Romania; 4 Doctoral School of Sport Science and Physical Education, Pitești University Center, National University of Science and Technology Politehnica Bucharest, 110253 Pitești, Romania; 5 High Institute of Sport and Physical Education of Sfax, University of Sfax, Sfax 3029, Tunisia; hamdi.chtourou@isseps.usf.tn; 6 Research Unit “Physical Activity, Sport and Health”, UR18JS01, National Observatory of Sport, Tunis 1003, Tunisia; n_souissi@yahoo.fr

**Keywords:** sleep, recovery, chronobiology, neuromuscular function, vigilance, reaction time

## Abstract

This study investigated the effects of a single-night sleep extension protocol on physical performance and cognitive function in physically active university students across different times of day. Using a within-subjects, counterbalanced crossover design, 24 physically active university students (17 males, 7 females; age: 22.7 ± 1.6 years) completed performance assessments under normal-sleep and sleep-extension conditions. Participants’ sleep was monitored via wrist actigraphy, and a comprehensive assessment battery comprising vertical jumps, Y-Balance tests, medicine-ball throws, 5 m shuttle-run tests, reaction-time tests, and digit-cancellation tests was administered at baseline (8 PM), morning (8 AM), and afternoon (4 PM). Sleep extension increased total sleep time by approximately 55 min (531.3 ± 56.8 min vs. 476.5 ± 64.2 min; *p* < 0.001, *d* = 0.91). Significant improvements were observed in 5 m shuttle-run performance at 8 AM (best distance: 102.8 ± 11.9 m vs. 93.3 ± 8.5 m, *p* < 0.001, *d* = 0.93; fatigue index: 13.1 ± 8.3% vs. 21.2 ± 9.5%, *p* < 0.001, *d* = 0.90), squat-jump heights (28.2 ± 8.0 cm vs. 26.3 ± 7.2 cm, *p* = 0.005, *d* = 0.25), simple reaction time (252.8 ± 55.3 ms vs. 296.4 ± 75.2 ms, *p* < 0.001, *d* = 0.66), and digit-cancellation performance (67.6 ± 12.6 vs. 63.0 ± 10.0 targets, *p* = 0.006, *d* = 0.40). Sleep extension significantly enhances both physical and cognitive performance in physically active individuals, with effects more pronounced during morning hours, partially attenuating typical circadian performance decline and establishing sleep extension as an effective, non-pharmacological strategy for optimizing performance capabilities.

## 1. Introduction

Sleep is a fundamental biological process impacting physiological restoration, cognitive function, and overall health [1]. For physically active individuals and athletes, optimal sleep facilitates recovery from training-induced stress, supports adaptation to training stimuli, and enhances subsequent performance capabilities [2]. Despite its recognized importance, evidence indicates that many athletes experience inadequate sleep duration and quality, potentially compromising performance potential and recovery capacity [3].

While the negative effects of sleep restriction on athletic performance have been extensively documented [4], research investigating the potential ergogenic effects of sleep extension represents a relatively novel area with promising implications for performance enhancement strategies [5]. Sleep extension involves systematically increasing sleep duration beyond an individual’s habitual patterns through earlier bedtimes, later wake times, or strategic napping protocols [6].

Preliminary evidence suggests positive influences of extended sleep duration on physical performance parameters. Mah et al. [7] demonstrated that collegiate basketball players extending sleep to 10 h per night for 5–7 weeks exhibited significant improvements in sprint performance, shooting accuracy, and subjective ratings of physical and mental well-being. Similarly, Schwartz and Simon [8] reported enhanced endurance performance and reduced perceived exertion following a six-day sleep-extension intervention in recreational runners. However, literature examining sleep-extension effects on complex neuromuscular tasks requiring high levels of coordination, balance, and explosive power remains limited [5].

The physiological mechanisms underlying potential performance benefits from sleep extension are multifaceted [9,10]. Extended sleep may enhance recovery through increased growth hormone secretion, facilitating tissue repair and protein synthesis [11]. Additionally, optimized sleep duration may improve neural functioning through enhanced synaptic plasticity and memory consolidation, potentially benefiting skill acquisition and execution [12,13]. From a cognitive perspective, extended sleep has been associated with improvements in attention, reaction time, and decision-making processes critical to athletic performance [5,9].

Time-of-day variations in performance represent another important consideration in athletic contexts. Circadian rhythms influence numerous physiological parameters, including core body temperature, hormone concentrations, and neuromuscular function, collectively affecting performance capabilities throughout the day [14]. Research has established diurnal variations in measures of muscle strength, power [15], and endurance, with peak performance typically observed in the late afternoon and early evening [16]. However, the interaction between sleep extension and time-of-day effects on performance remains largely unexplored, particularly regarding potential chronobiological phase shifts or alterations in the amplitude of performance rhythms [17].

A critical gap in the current literature concerns the potential differential effects of sleep extension across varying types of physical performance tasks and cognitive domains at different times of day. While studies have examined specific performance measures in isolation, comprehensive assessments integrating multiple physical and cognitive parameters within the same population are scarce [1,5]. Furthermore, the majority of research has focused on elite athletes, with limited attention to physically active non-elite populations who constitute a substantially larger demographic that could benefit from sleep-optimization strategies [3].

The time course of performance changes following sleep-extension interventions also warrants further investigation. Acute responses to extended sleep may differ from chronic adaptations, and understanding the temporal dynamics of these responses is crucial for developing practical recommendations for sleep management in athletic contexts [2,6]. Additionally, individual factors such as chronotype, habitual sleep patterns, and sleep efficiency may modulate the effectiveness of sleep-extension interventions, necessitating more nuanced analytical approaches that account for these variables [17].

Therefore, the present study aimed to investigate the effects of a single-night sleep-extension protocol on multiple domains of physical performance and cognitive function in physically active university students across different times of day. We hypothesized that sleep extension would enhance performance parameters, with the magnitude of improvement being more pronounced during morning assessments compared to afternoon and evening assessments. Furthermore, we anticipated that tasks requiring greater neuromuscular coordination and cognitive processing would demonstrate more substantial improvements following sleep extension compared to simpler strength-based tasks.

## 2. Materials and Methods

### 2.1. Participants

A priori power analysis using G*Power 3.1 software determined that 22 participants were required (effect size f = 0.25, α = 0.05, power = 0.80, within-factors design with 2 conditions and 3 time points). Thirty healthy undergraduate students were initially recruited from the High Institute of Physical Activity and Sports in Kef, Tunisia, with 24 participants (17 males, 7 females) meeting inclusion criteria. Participants had a mean age of 22.7 ± 1.6 years, height of 1.76 ± 0.09 m, body mass of 71.5 ± 11.3 kg, and body mass index (BMI) of 22.0 ± 2.2 kg⋅m^−2^. All exhibited neutral chronotype (Horne and Ostberg questionnaire: 47.9 ± 5.3 points) and good sleep quality (PSQI < 5; 3.0 ± 1.5 points).

Inclusion criteria encompassed physically active individuals (≥3 exercise sessions/week), ages 19–26 years, good health status (absence of diagnosed cardiovascular, metabolic, or sleep disorders; normal BMI 18.5–24.9 kg·m^−2^), and “moderate morning” or “no morning” chronotype [18]. Exclusion criteria comprised smoking history, cardiovascular disease, sleep disorders, PSQI ≥ 5 points [19], medications affecting sleep/performance, recent shift work, and recent trans-meridian travel. Data collection avoided examination periods. All participants provided written informed consent. The study protocol adhered to the Declaration of Helsinki and received approval from the Institutional Review Board (approval number: C-0009/2024).

### 2.2. Study Design

#### 2.2.1. Experimental Design

The study employed a within-subjects, counterbalanced crossover design with normal-sleep (NS) and sleep-extension (EXT) conditions. Participants completed both conditions in randomized order with a minimum four-day washout period. Testing occurred at identical times across conditions, with computer-generated randomization sequences that were concealed until assignment. Prior to the main protocol, participants underwent two familiarization sessions (48–72 h apart) to minimize learning effects. Data collection for each participant was completed within one month to control for seasonal variations.

#### 2.2.2. Experimental Protocol

**Figure 1:**
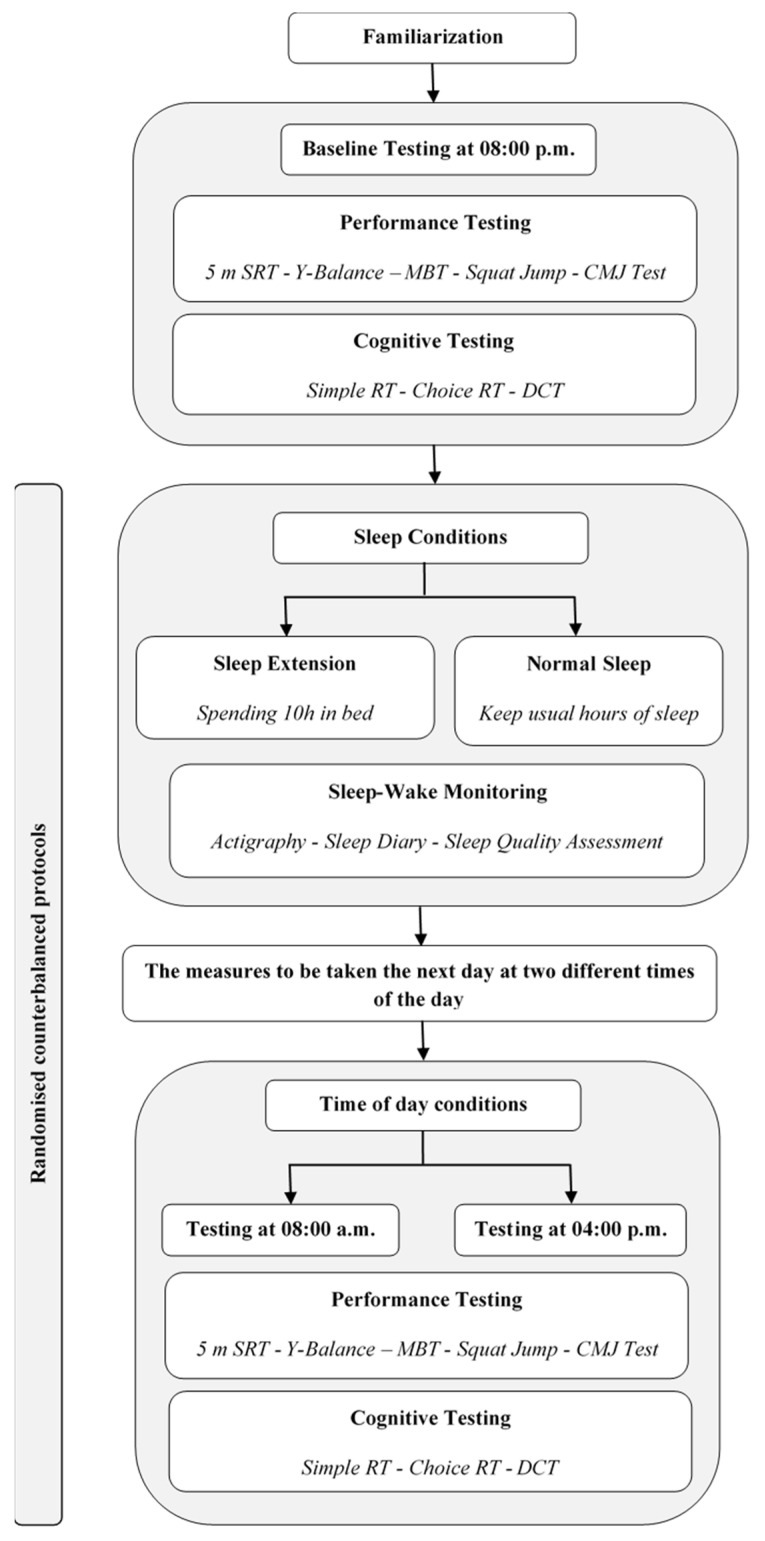
Schematic of the experimental protocol for the first study. SRT = shuttle-run test; CMJ = countermovement jump; RT = reaction time; DCT = digit cancellation test; MBT = medicine ball throw; Y-Balance = Y-Balance test. Time points: 08:00 = 8:00 AM; 04:00 = 4:00 PM.

Performance and cognitive assessments were conducted at three time points: 20:00 (8 PM), followed by 08:00 (8 AM) and 16:00 (4 PM) the next day under both sleep conditions. These time points were selected to capture circadian performance variations: 8 PM represents evening baseline, when core body temperature peaks; 8 AM captures the circadian trough period; and 4 PM represents the typical afternoon performance peak [14,20]. In NS, participants maintained habitual sleep schedules (bedtime: 22:30 ± 15 min, wake time: 07:00 ± 15 min). In EXT, sleep extension was achieved by advancing bedtime to 21:00 while maintaining wake time at 07:00, resulting in 10 h of time in bed, beginning approximately 30 min after evening testing [21]. The assessment battery was administered identically across all time points (Figure 1). Participants maintained normal dietary and activity patterns, abstaining from caffeine, alcohol, and nutritional supplements for 24 h pre-testing. Additional control measures included maintaining consistent meal timing, avoiding unusual physical exertion outside scheduled testing, and standardizing pre-test activities (no screens 1 h before sleep, consistent bedtime routine). Compliance was verified through 24 h recall questionnaires. Testing sessions occurred under standardized environmental conditions (temperature: 21 ± 1 °C; humidity: 45 ± 5%; barometric pressure: 755 ± 5 mmHg).

### 2.3. Assessment Procedures

#### 2.3.1. Sleep–Wake Monitoring

***Actigraphy:*** Sleep–wake patterns were assessed using wrist actigraphy (wGT3X-BT, ActiGraph LLC, Pensacola, FL, USA; 30 Hz) worn continuously on the non-dominant wrist. Actigraphy data were analyzed using ActiLife software (version 6.13.3) with the Cole–Kripke algorithm (sensitivity: 0.88, specificity: 0.80). Parameters extracted included total sleep time, time in bed, sleep efficiency, sleep-onset latency, wake-after-sleep onset, and awakenings. Sleep diary data were used to validate and supplement actigraphy measurements, particularly for determining precise sleep onset and offset times. Discrepancies between subjective reports and objective measurements were reconciled through individual participant consultation, consistent with established protocols for combining subjective and objective sleep assessments [22].

***Sleep Diary:*** Participants completed consensus sleep diaries each morning, recording bedtime, wake time, sleep latency, awakenings, and subjective sleep quality (5-point Likert scale). These data supplemented actigraphy measurements, particularly for sleep onset/offset times [22].

***Sleep Quality Assessment:*** Sleep quality was assessed using the French-validated Pittsburgh Sleep Quality Index (PSQI) [23], a 19-item self-report questionnaire evaluating seven domains (sleep quality, latency, duration, efficiency, disturbances, sleep medication use, and daytime dysfunction) over the previous month. The PSQI demonstrates good internal consistency (Cronbach’s α = 0.83) and test–retest reliability (r = 0.85).

#### 2.3.2. Performance Testing

All performance tests were conducted in a standardized order following a 10 min standardized warm-up consisting of low-intensity aerobic exercise, dynamic stretching, and sport-specific movements. A minimum 3 min recovery period was provided between each test to minimize fatigue effects [24]. Test order was counterbalanced across participants to control for potential sequence effects. The randomized test sequence, combined with standardized recovery periods, ensured that any residual carryover effects were distributed equally across conditions. All tests were administered by the same two researchers who were blinded to the experimental condition to prevent investigator bias.

***Explosive Power Tests****:* Vertical jumping ability was assessed via squat-jump (SJ) and countermovement-jump (CMJ) tests on a photoelectric cell (Optojump Microgate; 1000 Hz) [25]. For SJ, participants maintained 90° knee flexion for 3 s before jumping; for CMJ, they performed a rapid downward movement followed by a vertical jump. Participants kept hands on hips throughout both tests. The better of two trials (60 s recovery) was analyzed, with test–retest reliability of ICC = 0.92–0.97.

***Dynamic Balance Assessment****:* Dynamic balance was evaluated bilaterally using the Y-Balance test [26], measuring reach distances in anterior, posteromedial, and posterolateral directions while balancing on one leg. After four practice trials, three valid trials were performed for each leg. The composite score was calculated as the sum of the three reach distances normalized to leg length (anterior superior iliac spine to medial malleolus) × 100. The test demonstrates excellent reliability (intra-rater: ICC = 0.85–0.91; inter-rater: ICC = 0.99–1.00).

***Standing Backward Overhead Medicine Ball Throw****:* Upper-body power was assessed via backward overhead medicine ball throw [27]. Participants threw 3 kg (males) or 2 kg (females) medicine balls for maximum distance. The mean of three trials (90 s recovery) was analyzed (ICC = 0.92).

***5 m Shuttle-Run Test****:* Repeated sprint ability and fatigue resistance were assessed via the 5 m shuttle-run test (SRT) [28], comprising six 30 s sprints with 35 s recovery intervals. Distance was recorded manually with precision of ±1 m at each directional change during the 30 s shuttles. The following performance indices were recorded: (1) best distance (BD, m): the longest distance covered in any single 30 s shuttle; (2) total distance (TD, m): the sum of distances covered across all six shuttles; and (3) fatigue index (FI, %): calculated as [((shuttle1 + shuttle2)/2) − ((shuttle5 + shuttle6)/2)]/((shuttle1 + shuttle2)/2) × 100. This fatigue index formula has been validated for shuttle-run protocols and demonstrates good reliability (ICC = 0.89) in assessing anaerobic fatigue resistance [4,28]. RPE was recorded immediately after test completion. The 5 m SRT has demonstrated good reliability (CV = 2.8%) [28].

#### 2.3.3. Cognitive Testing

Cognitive assessments were performed in a quiet room with minimal distractions and controlled environmental conditions (temperature: 21 ± 1 °C; illumination: 500 lux). All computerized cognitive tests were conducted on the same laptop computer (Dell Latitude E7470, 14-inch screen, 1920 × 1080 resolution) with standardized screen brightness (75%) and contrast (50%). Participants were seated at a distance of 60 cm from the screen in an adjustable chair to ensure proper ergonomic positioning.

***Reaction Time Tests:*** Simple and choice reaction time were assessed using REACT V0.9 software [29]. For simple reaction time, participants responded to a single visual stimulus (green circle) across 20 trials, measuring mean reaction time (ms). For choice reaction time, participants discriminated between two stimuli (green vs. red circles) across 40 trials, measuring reaction time (ms) and accuracy (%). Both tests demonstrate good test-retest reliability (ICC = 0.87–0.92).

***Digit Cancellation Test:*** The digit cancellation test [30] assessed selective attention and processing speed, requiring participants to systematically scan rows of numbers and cross out all instances of specific target digits (3 and 7) within a 60 s time limit. Primary outcome measures were correct targets identified (number) and accuracy percentage. Participants were instructed to work as quickly and accurately as possible, scanning from left to right across each row. Performance was scored as correct targets identified (r = 0.84).

### 2.4. Statistical Analysis

Statistical analyses were performed using SPSS 29 (IBM Corp., Armonk, NY, USA) and Python 3.11.0 (Python Software Foundation, https://www.python.org/) with matplotlib 3.7.1, NumPy 1.24.3, and seaborn 0.12.2 packages. Data normality and variance homogeneity were verified using Shapiro–Wilk and Levene’s tests. A 2 × 3 (Condition [EXT, NS] × Time [8 PM, 8 AM, 4 PM]) repeated-measures ANOVA examined main effects and interactions. When sphericity was violated, Greenhouse–Geisser corrections were applied. Bonferroni-adjusted pairwise comparisons followed significant effects. Effect sizes were calculated as partial eta squared (*η*^2^_p_) for ANOVA effects (small: 0.01–0.05; medium: 0.06–0.13; large: ≥0.14) and Cohen’s d for pairwise comparisons (small: 0.2–0.49; medium: 0.5–0.79; large: ≥0.8). Relationships between sleep parameters and performance were examined using correlation analyses. Given the comparable sleep-extension effects on cognitive and physical performance between sexes in previous studies and our focus on acute sleep manipulation rather than sex-specific responses, male and female participants were analyzed together, with sex included as a covariate in all analyses [31]. Potential confounding variables (habitual sleep duration, physical activity levels, and BMI) were included as covariates in sensitivity analyses to ensure robustness of findings. Significance was set at *p* < 0.05, and 95% confidence intervals were calculated for primary outcomes.

## 3. Results

### 3.1. Participant Characteristics

Analysis of anthropometric data confirmed that participants (n = 24; 17 males, 7 females) had a mean age of 22.7 ± 1.6 years, height of 1.76 ± 0.09 m, body mass of 71.5 ± 11.3 kg, and BMI of 22.0 ± 2.2 kg⋅m^−2^. All participants exhibited neutral chronotype scores (47.9 ± 5.3 points; range: 44–58) and good sleep quality (PSQI: 2.9 ± 1.7 points; range: 0–4), confirming adherence to inclusion criteria.

### 3.2. Sleep Parameters

Analysis of actigraphic data confirmed successful implementation of the sleep-extension protocol. As shown in Table 1, participants achieved significantly greater total sleep time in the sleep-extension condition compared to normal sleep (531.3 ± 56.8 min vs. 476.5 ± 64.2 min; *p* < 0.001, *d* = 0.91), with time in bed also increasing significantly (563.6 ± 62.4 min vs. 514.2 ± 58.9 min; *p* < 0.001, *d* = 0.82). Sleep efficiency was marginally higher during sleep extension (95.1 ± 3.8% vs. 93.7 ± 4.3%; *p* = 0.045, *d* = 0.34), while sleep latency showed no significant difference.

### 3.3. Physical Performance Outcomes

#### 3.3.1. 5 m Shuttle-Run Test

Two-way repeated measures ANOVA revealed significant main effects of condition and time on best distance (BD), with a significant condition × time interaction (*p* = 0.012, *η*^2^_p_ = 0.174). Best distance was significantly greater in the EXT condition compared to NS at both 8 AM and 4 PM (*p* < 0.001), while no difference was observed at 8 PM baseline (Table 2).

**Figure 2:**
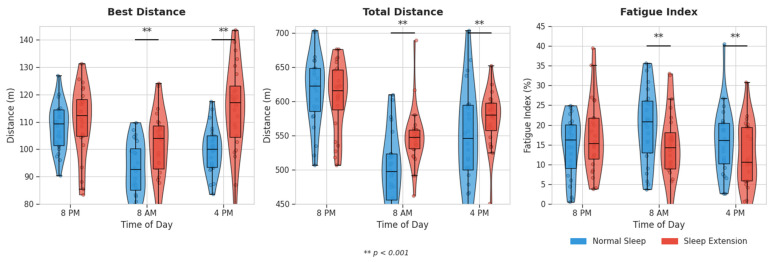
Effects of sleep extension vs. normal sleep on best distance and fatigue index during the 5 m shuttle-run test across different times of day. Individual data points are shown as colored circles (blue = Normal Sleep, red = Sleep Extension) with slight horizontal jitter to prevent overlapping and reveal the distribution of individual observations within each condition.

Similarly, total distance demonstrated significant improvements during EXT at 8 AM and 4 PM (*p* < 0.001), and the fatigue index was significantly lower in the EXT condition at these same time points (*p* < 0.001) (Figure 2).

#### 3.3.2. Jump Performance

Both the squat-jump and countermovement-jump heights showed significant main effects of condition and time, with significant interactions. Post hoc comparisons revealed significantly greater jump heights in the EXT condition at 8 AM and 4 PM for both jump types (*p* < 0.05), with small to medium effect sizes (*d*: 0.17–0.26).

#### 3.3.3. Medicine Ball Throw and Y-Balance Test

Standing backward overhead medicine-ball-throw distances showed significant effects of condition and time (*p* < 0.01), without a significant interaction. EXT yielded greater throwing distances at both 8 AM and 4 PM. Y-Balance test composite scores for both limbs revealed significant main effects of condition and time, with right limb scores significantly higher during the EXT condition at 4 PM (*p* = 0.007).

### 3.4. Cognitive Outcomes

#### 3.4.1. Reaction Time Tests

Simple reaction time demonstrated significant main effects of condition and time, with a significant interaction (*p* < 0.001, *η*^2^_p_ = 0.261). Sleep extension resulted in significantly faster reaction times at both 8 AM and 4 PM (*p* < 0.001), achieved during the EXT condition at both the morning and afternoon assessments (*p* < 0.01) with moderate to large effect sizes (*d*: 0.66–1.70). For choice reaction time test accuracy, higher accuracy was achieved.

#### 3.4.2. Digit Cancellation Test

**Figure 3:**
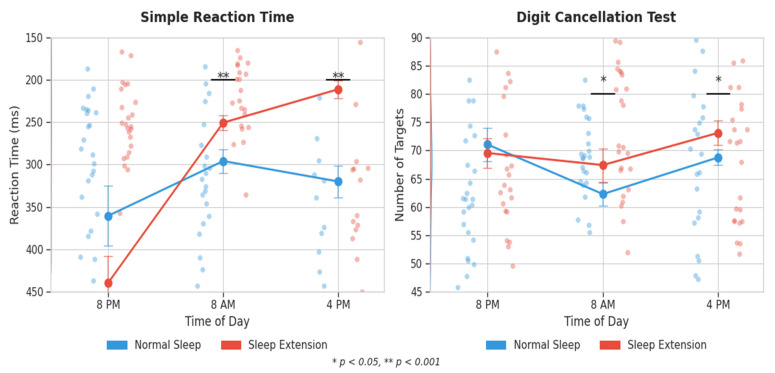
Effects of sleep extension vs. normal sleep on simple reaction time and digit cancellation test performance across different times of day. Values represent means ± standard error.

Digit cancellation test performance showed significant main effects of condition and time, with a significant interaction (*p* = 0.003, *η*^2^_p_ = 0.229). Participants identified significantly more targets in the EXT condition at both 8 AM and 4 PM (*p* < 0.01), with moderate effect sizes (Figure 3).

### 3.5. Time-of-Day Effects and Sleep–Performance Relationships

Significant time-of-day effects were observed across both conditions, with better performance generally occurring at 4 PM compared to 8 AM. The sleep-extension intervention attenuated these diurnal variations. Specifically, the amplitude of circadian performance variation was reduced from baseline levels, with the difference between peak (4 PM) and trough (8 AM) performance decreasing by approximately 23% for simple reaction time and 18% for shuttle-run best distance following sleep extension compared to normal-sleep conditions. Correlation analyses revealed significant positive associations between total sleep time in the EXT condition and improvements in 5 m shuttle-run best distance (r = 0.61, *p* = 0.001), reduced fatigue index (r = −0.56, *p* = 0.004), and enhanced simple reaction time (r = −0.63, *p* < 0.001). Sleep efficiency showed moderate positive correlations with CMJ height (r = 0.47, *p* = 0.020) and DCT performance (r = 0.45, *p* = 0.027).

## 4. Discussion

The present study investigated the effects of a single-night sleep-extension protocol on physical performance and cognitive function in physically active university students across different times of day. Our findings demonstrated that extending sleep duration by approximately 55 min significantly enhanced both physical and cognitive performance parameters, with improvements being more pronounced during morning assessments.

### 4.1. Effects on Physical Performance

The significant improvements in shuttle-run performance following sleep extension, evidenced by increased best distance and reduced fatigue index at 8 AM, align with previous research demonstrating the beneficial effects of extended sleep on intermittent sprint performance. These results expand upon Mah et al. [7], who reported significant improvements in sprint times following a 5–7-week sleep-extension intervention in collegiate basketball players. Our study extends this knowledge by demonstrating that even a single night of sleep extension can yield substantial performance benefits. The greater total distance and reduced fatigue index in the 5 m shuttle-run test suggest that sleep extension may enhance both anaerobic capacity and fatigue resistance, potentially through improved metabolic efficiency and enhanced recovery processes [2,6]. These findings are further supported by Mah et al. [7], who demonstrated that sleep extension in collegiate basketball players resulted in faster sprint times (16.2 ± 0.61 s vs. 15.5 ± 0.54 s, *p* < 0.001) and improved shooting accuracy. Similarly, Patrick et al. [32] found that acute sleep deprivation significantly impaired reaction time performance in university students, providing complementary evidence for the cognitive benefits we observed with sleep extension.

The observed improvements in explosive power, as measured by squat-jump and countermovement-jump heights, corroborate the findings of Schwartz and Simon [8], who reported enhanced power output following sleep extension in recreational athletes. These improvements, while statistically significant, were of smaller magnitude (*d*: 0.17–0.26) compared to the more substantial effects observed in shuttle-run parameters (*d*: 0.81–1.18), suggesting that tasks requiring greater neuromuscular coordination and sustained effort may be more sensitive to sleep manipulations than isolated explosive movements.

The significant enhancement in Y-Balance test performance indicates improved dynamic balance and neuromuscular control. Notably, Y-Balance improvements occurred specifically at 4 PM, likely reflecting the interaction between sleep-extension benefits and circadian enhancement of postural control during afternoon hours, when core body temperature and neuromuscular coordination typically peak [17]. This finding is particularly noteworthy, as dynamic balance requires integrated sensorimotor control and proprioceptive acuity [26]. Prior research has demonstrated that sleep restriction negatively impacts postural stability [1]; our findings extend the findings of this literature by demonstrating that sleep extension can positively influence these parameters, potentially through enhanced vestibular system function and improved sensorimotor integration.

### 4.2. Effects on Cognitive Performance

The pronounced improvements in simple reaction time and choice reaction time accuracy following sleep extension are consistent with previous investigations reporting enhanced cognitive processing following improved sleep quality and quantity [33]. The large effect sizes observed for reaction time improvements (*d*: 0.66–1.70) surpassed those for physical performance measures, suggesting that cognitive functions may be particularly responsive to sleep-extension interventions. The improvement in both speed and accuracy contradicts the typical speed–accuracy trade-off observed in many cognitive tasks, suggesting a fundamental enhancement in information processing efficiency [12].

The significant improvements in digit cancellation test performance (*p* = 0.006) further support the beneficial effects on selective attention and information processing speed. This finding expands upon work by Zimmerman et al. [34], who reported impaired cognitive performance following sleep restriction. Our findings suggest that sleep extension may optimize attentional resource allocation, potentially through enhanced prefrontal-cortex function and executive-control processes [35].

### 4.3. Time-of-Day Effects and Chronobiological Considerations

A novel finding was the interaction between sleep condition and time of day, wherein sleep extension partially attenuated but did not eliminate typical diurnal variations in performance. The persistent performance advantage at 4 PM compared to 8 AM across both sleep conditions aligns with established chronobiological principles regarding circadian rhythmicity of psychomotor performance [14,20]. However, the reduced performance gap between morning and afternoon assessments in the sleep-extension condition suggests that extended sleep may partially compensate for circadian disadvantages during morning hours.

The reduced sleep-extension benefits over increasing time awake reflect homeostatic sleep pressure accumulation. Extended sleep likely optimizes adenosine clearance and reduces baseline sleep pressure, providing greater cognitive and physical reserves during morning hours. However, as homeostatic sleep pressure gradually rebuilds throughout wakefulness, these benefits diminish, explaining the smaller effect sizes observed at 4 PM versus 8 AM [12,36].

### 4.4. Neurophysiological Mechanisms

While direct physiological biomarkers were not measured in this study, the observed performance patterns align with established sleep-extension effects on neural recovery processes. The time-dependent benefit reduction suggests homeostatic mechanisms rather than circadian phase shifts, though definitive mechanistic conclusions require future studies incorporating sleep architecture analysis and metabolic biomarkers [3,11].

### 4.5. Relationship Between Sleep Parameters and Performance

The observed correlations between total sleep time in the EXT condition and improvements in 5 m shuttle-run best distance (r = 0.61, *p* = 0.001), reduced fatigue index (r = −0.56, *p* = 0.004), and enhanced simple reaction time (r = −0.63, *p* < 0.001) provide strong evidence for a dose–response relationship between sleep duration and performance enhancement. Sleep efficiency showed moderate positive correlations with CMJ height (r = 0.47, *p* = 0.020) and DCT performance (r = 0.45, *p* = 0.027), highlighting the importance of both sleep duration and quality in determining performance outcomes, consistent with research by Lastella et al. [37].

### 4.6. Limitations

Some limitations warrant consideration. First, the acute nature of the sleep-extension protocol limits conclusions regarding potential adaptation effects with prolonged implementation. Second, the gender distribution was uneven (17 males and 7 females), potentially limiting generalizability across sexes. Additionally, all participants exhibited neutral chronotypes, precluding analysis of how chronotype might moderate the relationship between sleep extension and performance outcomes.

### 4.7. Practical Recommendations

Based on our findings, several practical recommendations emerge for athletes, coaches, and physically active individuals seeking to optimize performance through sleep management. First, individuals should aim to extend sleep duration beyond habitual patterns, particularly before morning competitions or events requiring high cognitive demand. A target increase of 45–60 min appears sufficient to elicit meaningful performance benefits. Second, prioritizing both sleep duration and quality is essential; our results demonstrated significant correlations between sleep efficiency and performance outcomes. Third, individuals who typically perform suboptimally in the morning should consider sleep extension as a countermeasure to morning performance decrements. Finally, monitoring sleep patterns using wearable technology or sleep diaries can provide valuable insights into individual sleep needs and the effectiveness of sleep-extension strategies.

## 5. Conclusions

This study provides compelling evidence that a single night of sleep extension significantly enhances multiple domains of physical performance and cognitive function in physically active individuals, with effects being more pronounced during morning hours. The differential magnitude of improvements across performance parameters suggests task-specific sensitivity to sleep manipulation, with complex, sustained efforts and cognitive functions showing the greatest benefit. The partial attenuation of diurnal performance variations following sleep extension holds relevance for morning competitions. The observed benefits from approximately 55 min of additional sleep align with dose–response research demonstrating meaningful cognitive and physical improvements with sleep extensions of 46–113 min in young adults. This duration likely optimizes sleep architecture by increasing the slow-wave and REM sleep phases, which are critical for physiological and cognitive recovery. These findings establish sleep extension as an effective, non-invasive strategy to optimize performance capabilities across diverse athletic contexts and provide a scientific rationale for implementing sleep-extension protocols in athletic and academic settings where optimal performance is required.

## Figures and Tables

**Table 1:** Sleep parameters for normal-sleep and sleep-extension conditions.

Parameter	Normal Sleep	Sleep Extension	Mean Difference [95% CI]	*p*-Value	Cohen’s *d*
Time in bed (min)	514.2 ± 58.9	563.6 ± 62.4	49.4 [39.3, 59.5]	<0.001	0.82
Total sleep time (min)	476.5 ± 64.2	531.3 ± 56.8	54.8 [43.3, 66.3]	<0.001	0.91
Sleep efficiency (%)	93.7 ± 4.3	95.1 ± 3.8	1.4 [0.1, 2.7]	0.045	0.34
Sleep latency (min)	22.6 ± 16.8	19.5 ± 13.2	−3.1 [−6.9, 0.7]	0.105	0.21

Data presented as means ± standard deviations. CI = confidence interval.

**Table 2:** Effects of sleep extension on performance and cognitive outcomes across time of day.

Variable		Time	Normal Sleep	Sleep Extension	Condition Effect	Time Effect	Interaction Effect
5 m SRT	Best Distance (m)	8:00 PM	108.5 ± 10.3	109.7 ± 10.5	*p* < 0.001 *η*^2^_p_ = 0.561	*p* < 0.001 *η*^2^_p_ = 0.591	*p* = 0.012 *η*^2^_p_ = 0.174
		8:00 AM	93.3 ± 8.5	102.8 ± 11.9 **			
		4:00 PM	98.2 ± 9.8	110.6 ± 11.4 **			
	Total Distance (m)	8:00 PM	591.2 ± 57.1	598.3 ± 58.2	*p* < 0.001 *η*^2^_p_ = 0.598	*p* < 0.001 *η*^2^_p_ = 0.567	*p* = 0.026 *η*^2^_p_ = 0.147
		8:00 AM	510.3 ± 49.6	547.3 ± 43.2 **			
		4:00 PM	539.0 ± 55.3	586.0 ± 45.3 **			
	Fatigue Index (%)	8:00 PM	15.8 ± 7.6	15.1 ± 7.7	*p* < 0.001 *η*^2^_p_ = 0.431	*p* < 0.001 *η*^2^_p_ = 0.457	*p* = 0.006 *η*^2^_p_ = 0.199
		8:00 AM	21.2 ± 9.5	13.1 ± 8.3 **			
		4:00 PM	16.5 ± 8.4	10.2 ± 7.0 **			
	RPE	8:00 PM	7.5 ± 0.6	7.2 ± 0.5	*p* = 0.005 *η*^2^_p_ = 0.290	*p* < 0.001 *η*^2^_p_ = 0.417	*p* = 0.166 *η*^2^_p_ = 0.075
		8:00 AM	8.4 ± 0.6	7.8 ± 0.4 **			
		4:00 PM	8.0 ± 0.5	7.5 ± 0.3 *			
Y-Balance Test	Right (%)	8:00 PM	115.5 ± 13.8	115.9 ± 14.0	*p* = 0.010 *η*^2^_p_ = 0.255	*p* = 0.031 *η*^2^_p_ = 0.140	*p* = 0.158 *η*^2^_p_ = 0.077
		8:00 AM	109.3 ± 12.2	112.8 ± 12.9			
		4:00 PM	110.1 ± 13.4	115.0 ± 12.6 *			
	Left (%)	8:00 PM	103.5 ± 19.7	103.8 ± 19.5	*p* = 0.031 *η*^2^_p_ = 0.186	*p* = 0.071 *η*^2^_p_ = 0.109	*p* = 0.203 *η*^2^_p_ = 0.067
		8:00 AM	97.7 ± 16.3	99.5 ± 16.8			
		4:00 PM	98.1 ± 17.0	101.7 ± 16.3 *			
Medicine Ball Throw (m)		8:00 PM	9.8 ± 2.0	9.9 ± 2.1	*p* = 0.008 *η*^2^_p_ = 0.269	*p* = 0.019 *η*^2^_p_ = 0.159	*p* = 0.121 *η*^2^_p_ = 0.088
		8:00 AM	8.9 ± 2.2	9.4 ± 2.2 *			
		4:00 PM	9.8 ± 2.3	10.5 ± 2.4 *			
Squat Jump Height (cm)		8:00 PM	28.5 ± 8.1	29.0 ± 8.4	*p* = 0.003 *η*^2^_p_ = 0.329	*p* = 0.002 *η*^2^_p_ = 0.231	*p* = 0.029 *η*^2^_p_ = 0.143
		8:00 AM	26.3 ± 7.2	28.2 ± 8.0 *			
		4:00 PM	27.7 ± 7.3	29.6 ± 7.5 *			
CMJ Height (cm)		8:00 PM	30.4 ± 8.7	30.6 ± 8.6	*p* = 0.006 *η*^2^_p_ = 0.287	*p* = 0.010 *η*^2^_p_ = 0.183	*p* = 0.043 *η*^2^_p_ = 0.128
		8:00 AM	28.0 ± 8.1	29.4 ± 8.6 *			
		4:00 PM	29.3 ± 8.7	31.1 ± 9.0 *			
Simple RT (ms)		8:00 PM	362.8 ± 159.8	359.7 ± 151.9	*p* < 0.001 *η*^2^_p_ = 0.504	*p* < 0.001 *η*^2^_p_ = 0.415	*p* < 0.001 *η*^2^_p_ = 0.261
		8:00 AM	296.4 ± 75.2	252.8 ± 55.3 **			
		4:00 PM	334.1 ± 91.2	209.5 ± 47.8 **			
Choice RT Accuracy (%)		8:00 PM	95.1 ± 2.9	95.8 ± 2.6	*p* = 0.004 *η*^2^_p_ = 0.310	*p* = 0.008 *η*^2^_p_ = 0.189	*p* = 0.124 *η*^2^_p_ = 0.087
		8:00 AM	96.4 ± 2.4	97.9 ± 1.6 *			
		4:00 PM	95.2 ± 3.2	98.0 ± 1.4 **			
DCT (targets)		8:00 PM	70.9 ± 15.3	70.3 ± 14.7	*p* = 0.002 *η*^2^_p_ = 0.360	*p* = 0.009 *η*^2^_p_ = 0.186	*p* = 0.003 *η*^2^_p_ = 0.229
		8:00 AM	63.0 ± 10.0	67.6 ± 12.6 *			
		4:00 PM	68.0 ± 10.3	73.3 ± 11.0 *			

Data presented as means ± standard deviations. SRT = shuttle-run test; RPE = rating of perceived exertion; CMJ = countermovement jump; RT = reaction time; DCT = digit cancellation test; *η*^2^_p_ = partial eta squared. * *p* < 0.05, ** *p* < 0.001 for post hoc comparisons between conditions.

## Data Availability

The data presented in this study are available on request from the corresponding author. The data are not publicly available due to confidentiality.

## Abbreviations

The following abbreviations are used in this manuscript:

ANOVA	analysis of variance
BD	best distance
BMI	body mass index
CI	confidence interval
CMJ	countermovement jump
CRT	choice reaction time
CV	coefficient of variation
DCT	digit cancellation test
EXT	sleep extension
FI	fatigue index
ICC	intraclass correlation coefficient
LLC	Limited Liability Company
min	minutes
ms	milliseconds
NS	normal sleep
PSQI	Pittsburgh Sleep Quality Index
REM	rapid eye movement
RPE	rating of perceived exertion
RT	reaction time
SJ	squat jump
SRT	shuttle-run test
TD	total distance
*η*^2^_p_	partial eta squared
